# Chemotypic variation in terpenes emitted from storage pools influences early aphid colonisation on tansy

**DOI:** 10.1038/srep38087

**Published:** 2016-11-28

**Authors:** Mary V. Clancy, Sharon E. Zytynska, Matthias Senft, Wolfgang W. Weisser, Jörg-Peter Schnitzler

**Affiliations:** 1 Helmholtz Zentrum München, Institute of Biochemical Plant Pathology, Research Unit Environmental Simulation (EUS), Neuherberg, Germany; 2 Technische Universität München, Terrestrial Ecology Research Group, Department of Ecology and Ecosystem Management, School of Life Sciences Weihenstephan, Freising, Germany

## Abstract

Tansy plants (*Tanacetum vulgare* L.) exhibit high chemical variation, particularly in mono- and sesquiterpenes that are stored in specialised glands on the plant surface. In the present work we investigated the effects of terpene chemotypes on *Metopeurum fuscoviride*, an aphid species specialised on tansy, and their tending ants, at the field scale. Previous studies have chemotyped tansy by assessing dominant compounds; here we propose a method of chemotyping using all volatile compounds that are likely emitted from the storage glands. The analysis is based on two extraction methods: GC-MS analysis of leaf hexane extracts and SBSE analysis of headspace emissions. In an initial screening we identified the subset of compounds present in both chemical patterns, labelled as ‘compounds likely emitted from storage’. In a large field survey we could show that the putative chemotypic emission pattern from storage pools significantly affected the early aphid colonisation of tansy. Moreover, the statistical analyses revealed that minor compounds exerted a stronger influence on aphid and tending-ant presence than dominant compounds. Overall we demonstrated that within the enormous chemotypic variation of terpenes in tansy plants, chemical signatures of volatile terpenes can be related to the occurrence of insects on individual plants in the field.

Plant secondary metabolites comprise extremely diverse groups of compounds that can be found across the plant kingdom. These chemicals exhibit high levels of variation in form and abundance both across and within plant families, as well as species. Biogenic volatile organic compounds (VOCs) are natural compounds that have high vapour pressure/high volatility, and consist of a large range of structures^1^. Volatile compounds play an important role in the relationship between plants and insects, and influence the interactions of many species^2^, including the defence of plants against their herbivores. As plants are sessile organisms, they have had to develop several direct and indirect defence mechanisms. Herbivores can be challenged by plants in several ways. These can include so-called direct defences, e.g. the plant producing toxic compounds (e.g. cardenolides)^3^ and digestibility reducers (e.g. proteinase inhibitors)^4^, as well as making itself unsuitable for the herbivore’s survival or reproduction^5^. VOCs can be involved in an indirect method of defence whereby plants utilise them as infochemicals to attract herbivore enemies (which can be thought of as a ‘call for help’)^6^,^7^,^8^.

A major source of chemical variation in plants comes from terpenes (syn. terpenoids, isoprenoids) which constitute a large class of plant secondary metabolites^9^. So far approximately 25,000 structures have been reported. Terpenes are initially produced by terpene synthases, which are often able to produce multiple compounds from single substrates^10^,^11^. Subsequently these terpene backbones are modified in multiple ways, generating the large number of chemical structures^1^,^12^. Terpenes are known to be involved in both the direct and indirect defence mechanisms of plants^13^, and can be released from plants following disruption of reservoir glands, or by active synthesis triggered by abiotic and biotic stress^14^ (Fig. 1). Many plant species possess reservoir glands in which they store terpenes, for example: external secreting trichromes are found in the *Lamiaceae*^15^ and *Solanaceae*^16^ families, while conifers have internal resin canals and ducts in their needles^17^. The metabolic mixtures of these stored terpenes are highly inter- and intraspecific, and can be used for the characterisation of species and phenotypes by chemotyping^18^. The terpene profile of a plant (chemotype) has been found to serve a number of ecological roles^19^ with respect to interactions among organisms. For example, a chemotype of the Australian tea tree (*Melaleuca alternifolia*) containing high levels of terpinolene was consumed less by herbivores than a chemotype of this species containing high levels of eucaliptol^20^.

Tansy (*Tanacetum vulgare* L.; Asteraceae) is a well-known aromatic perennial herb that is native to Eurasia and is an established invasive in North America. It is highly diverse in terpene content^21^. Moreover, various abiotic and biotic stressors have been shown to affect the VOC profile leading to differences between chemotypes^22^,^23^. So far, few studies have investigated the distribution of tansy chemotypes on a small scale, but plant chemotypes are known to vary considerably even within a single field site^22^,^24^.

Tansy is colonised by several species of specialised aphids, the most common being the pink tansy aphid, *Metopeurum fuscoviride* Stroyan (Aphididae), that is generally tended by ants^25^. These aphids exhibit a metapopulation structure on tansy, with dispersal among plants limited to the few generations (or weeks) when winged aphids are present^26^. The aphids interact with their tending ant species, with other specialised tansy aphid species and the myriad of natural enemies to form a metacommunity. Previous work^24^,^27^,^28^ has shown that tansy chemotypes dominated by camphor, β-thujone, artemisia ketone and borneol can influence the community of associated invertebrates^18^,^29^. However, many of these studies classified the plants into chemotypes based on the dominant terpenes, yet often it is the whole ‘blend’ of a plant’s emitted terpenes that has been found to influence plant-insect interactions^30^. Tansy has both tectorial and glandular trichromes on its leaf surface^31^. Mono- and sesquiterpenes are stored in the oil reservoirs of the trichomes, while various classes of compounds (e.g. green leaf volatiles (GLVs), benzenoids (BZs), sesquiterpenes (SQTs), monoterpenes (MTs)) can be induced through abiotic and biotic stresses, i.e. herbivory. Compounds that are stored in these specialised structures are released either by temperature-dependent evaporation or upon mechanical rupture of the glands. Stress-induced compounds however, are formed by active biosynthesis and are immediately emitted. When not mechanically disrupted, the volatile compounds accumulated and stored in the glands are only emitted at low rates. Differing vapour pressures and chemical properties (for example Henry’s law constants) result in some compounds not able to diffuse out from oil reservoirs under ambient conditions (i.e. not in extreme temperatures)^32^. The compounds that are constitutively emitted from glands of undamaged leaves are of ecological relevance as they form the volatile chemotype ‘visible’ to passing herbivores and herbivore enemies. In turn, as a plant is colonised, herbivore feeding leads to an increase in the release of volatiles and production of stress-induced compounds, resulting in a different volatile blend than from a healthy plant^33^,^34^,^35^.

The aim of the present work was to assess the chemotypic variation of tansy plants in a single field site (less than 1 km^2^) and determine chemotype-specific differences in stored and/or emitted volatiles, and how these differences can influence aphid colonisation of the plants. As ant mutualists are known to be important for successful aphid colonisation, we also aimed to explore whether or not plant compounds influenced the presence of two aphid-tending ant species: *Lasius niger* and *Myrmica rubra*. From our data analysis we suggest that by excluding stress-induced compounds from the emission spectrum the effects of different plant chemotypes on the associated invertebrate community can be determined.

## Results

### Comparison of VOC pattern in hexane extracts and headspace collections

**Figure 1:**
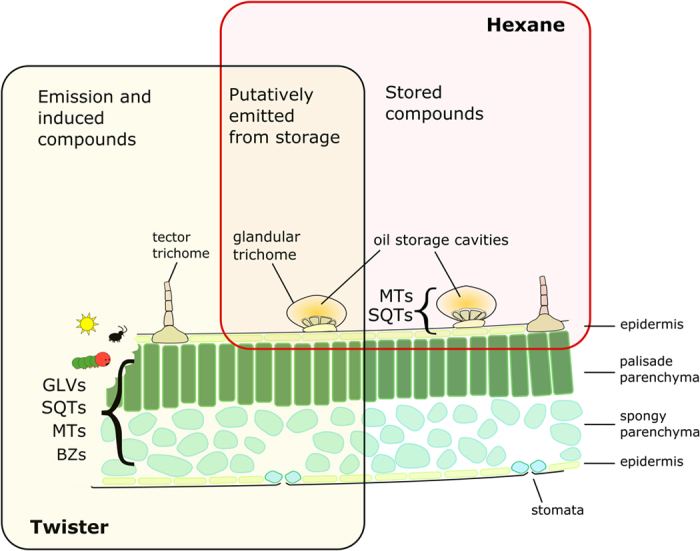
Schematic image of plant tissue in which terpenes and other VOCs are stored and/or synthesised. All VOCs that are emitted from the leaf were analysed in headspace collections using the SBSE (stir bar sorptive extraction) ‘twister’ method. The hexane extraction method includes lipophilic compounds that are stored in specialised compartments. Compounds that are likely emitted from storage pools are found in the overlap of compounds between the hexane and twister methods.

**Figure 2:**
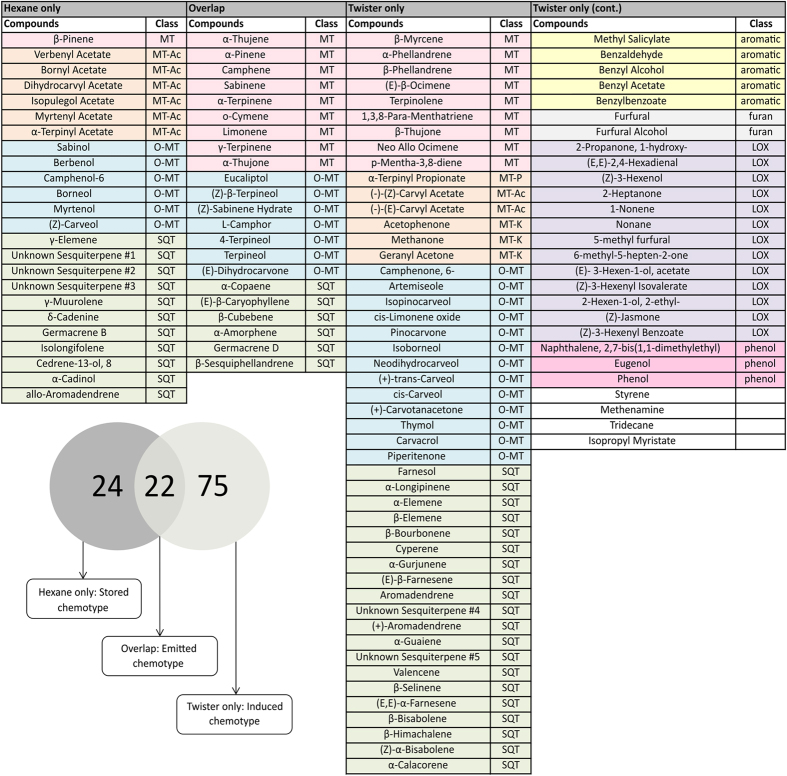
Compounds identified in the hexane and SBSE (stir bar sorptive extraction) VOC analysis method. From 28 tansy plants, 22 compounds were identified in the metabolite pattern using both methods. These overlap compounds were tentatively named the ‘likely emitted from storage’ chemotype. Twenty-four compounds were identified in the hexane ‘stored compounds’ fraction alone, whereas 75 volatiles could be identified exclusively in the headspace fraction alone. The Venn plot represents a visualisation of this.

The aim of this initial experiment with 28 tansy plants was to comparatively analyse the spectrum of stored compounds and volatiles released into the headspace. Volatile compounds emitted by the plant may originate from the storage glands on the surface of the leaves, or can be synthesised in response to various stresses including herbivory, environmental stressors, infection etc. (Fig. 1). Overall a total of 121 compounds could be identified by GC-MS analysis, which were ordered to nine different chemical classes (Fig. 2). Forty-six compounds were detected in the hexane extraction of the 28 analysed plants, in contrast to the 97 compounds identified in the headspace of bagged plants, with 22 compounds being detected by both methods. Compounds found in the hexane extracts were mainly mono- and sesquiterpenes. A much wider range of compound groups could be detected in the headspace, including volatile benzenoids and products from the lipoxygenase pathway, as well as terpenes that are commonly induced in response to biotic and abiotic stresses. The 22 compounds common to both the SBSE and hexane VOC analysis methods are compounds that are stored in the leaf but are also detectable in the emission profile of tansy, as such, this group of compounds was named ‘likely emitted from storage’ (see Fig. 1). This group comprises several common monoterpenes including sabinene, limonene and camphor, as well as sesquiterpenes including (*E*)-β-caryophyllene and β-cubebene. The compounds found only in the hexane profile were dubbed the ‘stored’ fraction, as the majority of these compounds are constitutively stored and not emitted; the compounds found only in the SBSE analysis were called ‘headspace only’ compounds.

Whereas only 28 plants in the field were analysed using the SBSE method, all plants in the field were assessed using the hexane method. All compounds identified in the hexane extracts of all 176 plants were present in the subset of plants analysed using the SBSE method, meaning that no compounds were excluded simply because they were not present in the plants used for the headspace analysis.

### Stability of the stored ‘terpene’ chemotype

To analyse the stability of the intrinsic compound pattern in hexane extracts we propagated seven tansy plants in the greenhouse and analysed the concentration and compound spectrum in five clonal plants of each genotype. The same compounds were detected among clones.

**Figure 3:**
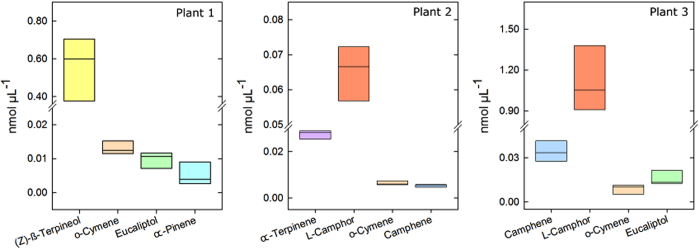
Example of the variation in terpene concentration in three genotypes of three clonally propagated, greenhouse-reared tansy plants (n = 5, ±SD).

Globally, we observed a variation in the concentrations of individual terpenes of approximately 15% between clones within a genotype, indicating that chemotypes of the 176 plants collected in the field can be assigned as, allowing for technical and handling variation, there is a high level of chemical stability in each plant (Fig. 3).

### Field survey of tansy chemotypes

#### Chemotype-level analysis

**Figure 4:**
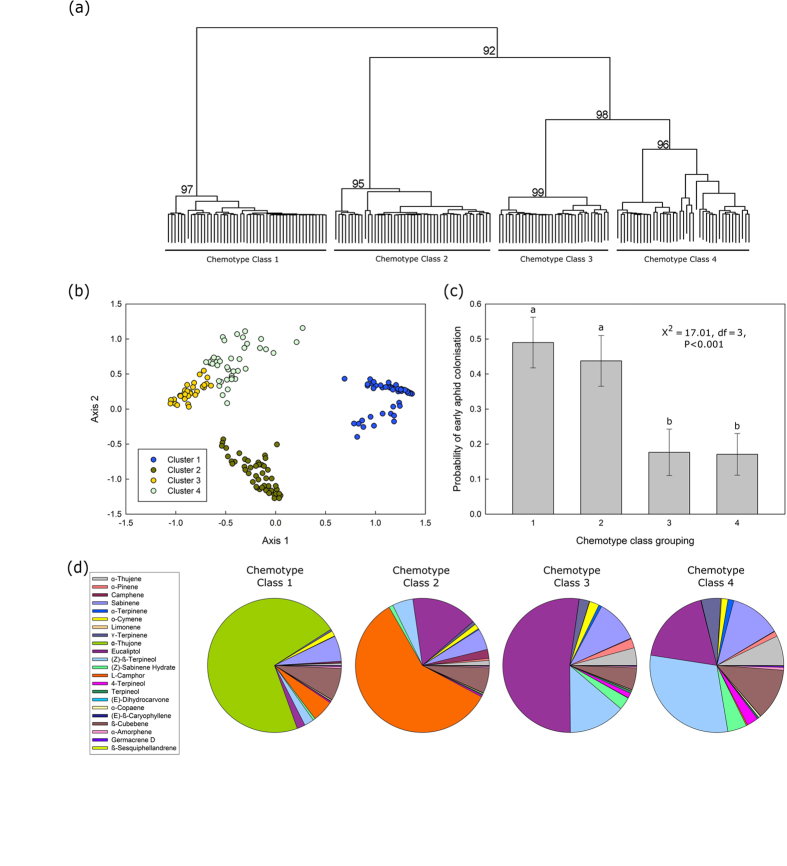
Results of chemotype clustering. (**a**) Hierarchical Cluster Analysis of ‘likely emitted from storage’ compound concentrations across 176 individual plants. Four main groups were identified and labelled Chemotype Classes 1–4, (**b**) shows a Multidimensional Scaling Diagram (MDS) plot with two artificial axes where each plant is placed as one point, with similar samples being plotted together, (**c**) shows that the probability of early aphid colonisation is significantly higher (P < 0.001) on plants with chemotype classes 1 and 2, and (**d**) shows real examples of all 4 chemotype classes.

In the main experiment we analysed the compound pattern in hexane extracts of 176 tansy plants growing on the field plot (Table S1). Using the introduced classification from above (Fig. 2), i.e. dividing terpenes between ‘stored’ and ‘likely emitted from storage’ VOCs, we statistically classified the 176 plants into chemotype classes using compounds from the ‘likely emitted from storage’ fraction. The plants clustered into four major classes (Fig. 4a,b). Three of the four groups were characterised by particular dominant compounds (i.e. compounds that make up over 40% of the whole compound profile), with class 1 dominated by α-thujone, class 2 by camphor, and class 3 by eucaliptol. Class 4 was characterised by a slight dominance (i.e. not clearly dominating the compound profile) of (*Z*)-β-terpineol, however overall the plants in this class are represented by a mixture of compound concentrations (Fig. 4d).

Plants with similar chemotype profiles were not geographically clustered, instead plants with similar chemotype profiles were distributed randomly across the field site (chemical distance to geographic distance: Mantel test, r = 0.05, P = 0.112, Fig. S2). In the tansy aphid life-cycle, the main dispersal period occurs at the end of May/early June (early aphid colonisation) when winged morphs first appear and most plants are unoccupied, i.e. aphids are free to choose among host plants^26^,^36^. Winged aphids are only produced for a few weeks, after which new colonisations of empty plant patches occurs by walking unwinged aphids (late colonisation, from late June). The early colonisation of pink tansy aphids varied across these four chemotype classes (Χ^2^ = 17.01, df = 3, P < 0.001). In classes 1 and 2, 43–49% of the young plants were colonised by aphids whereas only 17% of the plants in classes 3 and 4 were colonised (Fig. 4c). There was no effect of plant chemotype class on the new colonisation of (still) empty plant patches at the end of the season, when colonisation almost exclusively occurs by walking aphids (Χ^2^ = 5.77, df = 3, P = 0.124), or on the presence of either of the tending ant species before aphids colonised the plants (*Lasius niger*: Χ^2^ = 3.24, df = 3, P = 0.356; *Myrmica rubra*: Χ^2^ = 3.06, df = 3, P = 0.382). When the plants were clustered in the same way using the overall ‘stored’ terpenes rather than the terpenes ‘likely emitted from storage’, we observed no effect of chemotype groupings on any aphid or ant response variables (P > 0.05).

#### Compound-level analysis

**Figure 5:**
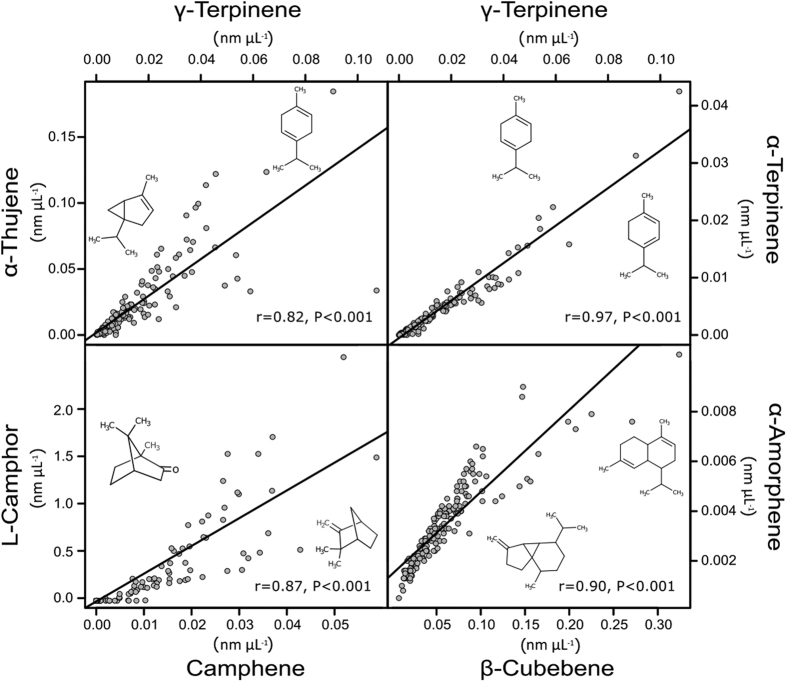
Relationship between top eight correlated terpenes in the 176 tansy plants, indicating a tightly linked biosynthesis. Panels (**a**), (**b**), (**c**) and (**d**) show the correlations between γ-terpinene and α-thujene, γ-terpinene and α-terpinene, camphene and L-camphor, and β-cubebene and α-amorphene respectively.

Independent of the classification into chemotypes we performed a correlation analysis (Fig. S1), comparing the concentration of each terpene ‘likely emitted from storage’ to one other. The results revealed several pairs of terpenes which showed a high correlation of concentrations among the 176 plants (Fig. 5). Some obviously chemically related compounds correlated within a plant, for example α- terpinene and γ-terpinene, which is an indication for a tightly linked biosynthesis. γ-Terpinene concentrations were also strongly correlated to the concentrations of α-thujene, α-terpinene and 4-terpineol, while camphene correlated to L-camphor. Another cluster of highly correlated concentrations was (*Z*)-sabinene hydrate to (*Z*)-β-terpineol and 4-terpineol. Among sesquiterpenes, (*E*)-dihydrocarvone was correlated to α-copaene, and β-cubebene to α-amorphene.

Aiming to understand the role of less-abundant terpenes in the odour profiles on the response variables, we applied a Bayesian model averaging approach with each compound as its own explanatory variable. After controlling for the effect of known covariates (i.e. plant size and accessibility from outside the field site) (Senft *et al*., unpublished data) we found that the ‘likely emitted from storage’ terpenes explained significant variation in all the variables. Those terpenes with the highest effect in the modelling were not the most dominant (Pearson correlation: r = −0.055, P = 0.811; Table S2). Further, compounds expressing a higher degree of variation among the different plants did not have a stronger effect (r = −0.028, P = 0.902; Table S2), indicating that the statistical analysis we used is not biased toward retaining chemicals that are either most dominant or more variable in the plant odour profiles.

**Figure 6:**
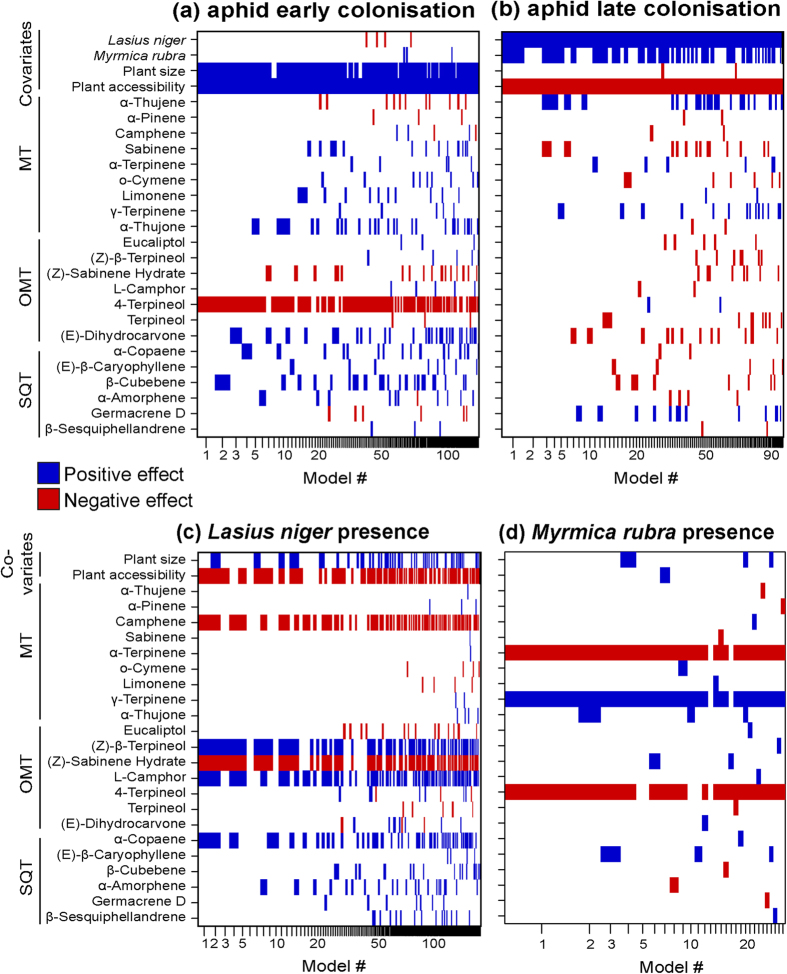
Results of Bayesian Model Averaging analyses evaluating the effect of plant emitted compounds on (**a**) early colonisation of aphids (during main dispersal phase), (**b**) late colonisation of pink tansy aphids (after main dispersal phase), (**c**) presence of *Lasius niger* before aphid colonisation, and (**d**) presence of *Myrmica rubra* before aphid colonisation. The width of each column is proportional to the model’s posterior probability, all retained models have posterior probabilities within a factor of 1/20 of that of the best model. The proportion of filled space is equivalent to the posterior effect probability of the explanatory variable, with blue indicating a positive effect and red a negative effect. Full model details can be found in the Table S2a,b,c and d.

Early aphid colonisation was more frequent on larger plants than on smaller plants and those that were more accessible from outside the field, indicating immigration by winged aphids. Colonisation was strongly negatively associated with 4-terpineol, and positively with α-thujone, (*E*)-dihydrocarvone, α-copaene and β-cubebene (Fig. 6a, Table S3a). After the main dispersal phase, late aphid colonisation (late June until August) was more likely on less accessible plants, indicating within-field dispersal, and was strongly enhanced by the presence of mutualistic ants (*Lasius niger* and *Myrmica rubra*) (Fig. 6b; Table S3b). Late aphid colonisation was positively associated with higher concentrations of α-thujene and γ-terpinene, but lower concentrations of sabinene (Fig. 6b; Table S3b). Ant mutualists are important for the aphid colonisation of tansy plants, particularly after the main dispersal phase, and the influence of plant terpenes on the presence of ants before aphid colonisation varied among the two ant species (Fig. 6c,d). In particular, *L. niger*, the ant species most beneficial for the aphids, was found most often on plants containing less (*Z*)-sabinene hydrate and camphene, but with more (*Z*)-β-terpineol and α-copaene (Fig. 6c; Table S3c), whereas *M. rubra* was found on plants with higher γ-terpinene and (*E*)-β-caryophyllene, and lower α-terpinene and 4-terpineol contents (Fig. 6d; Table S3d).

The statistical analysis revealed that stored compounds not present in the putative odour profile had less effect on the aphids and ants than the ones ‘likely emitted from storage’ (higher posterior probabilities were obtained from the compounds ‘likely emitted from storage’ models than the stored compound models; Tables S4a–d). Nevertheless we saw some weak statistical effects of stored compounds on the associated community. Early aphid colonisation by the pink tansy aphid was negatively associated with the presence of α-terpinyl acetate (Table S4a), whereas there was little effect of any other terpene on late plant colonisation; although there was a negative association with unknown sesquiterpenes (Table S4b). *Lasius niger* ants were observed more often on tansy plants with higher concentrations of germacrene B but lower concentrations of verbenyl acetate and berbenol (Table S4c). *Myrmica rubra* ants were more often observed on plants with higher concentrations of bornyl acetate and allo-aromadendrene, but lower content of α-cadinol (Table S4d).

## Discussion

We showed that the ‘putative’ chemotypic emission profiles of individual tansy plants influence the colonisation of aphids (*Metopeurum fuscoviride*) in the early part of the season, with almost half of the plants in classes 1 and 2 of our four chemotype classes being colonised by aphids, while less than a fifth were colonised in classes 3 and 4. Moreover, we could demonstrate that individual terpenes, particularly those which did not dominate the chemical composition, influenced the presence of ants on the plants before aphids were present and subsequently colonised the plants. Lastly, we were able to show that the profile of terpenes ‘likely emitted from storage’ is stable across clonally propagated tansy plants, which means they are suitable for use in manipulation experiments to further understand the role of constitutively expressed tansy chemotypes on the tansy-aphid-ant system.

While tansy is well known for its chemotypic variation^24^, the methods used to analyse VOCs and to assign the chemotypes vary greatly in the literature. Previous reports assessed tansy chemotypes by using essential oil analysis^37^, analysing only a subset of potential compounds^27^, or by performing the analysis of terpenes after air-drying of the samples^28^. Therefore the literature information is incomplete, and further, due to the possible loss of compounds either by dilution or inappropriate extraction of volatile compounds, the interpretation of the chemotypic importance of tansy in plant-insect interactions is difficult. In the present work we chose to perform hexane extractions using freshly shock frozen (into dry-ice immediately upon harvest) leaf material that was then stored at −80 °C prior to extraction.

We were able to identify 46 compounds in the hexane extracts by GC-MS analysis according to database searches, retention time indices, and comparison with authentic standards. By comparing these compounds to volatile compounds collected in the headspace, we were able to identify those volatile terpenes that are stored in the glands and are likely emitted – i.e. the ‘blend’ of a plant independent of abiotic/biotic stress induced compounds such as GLVs, mono- (MTs) and sesquiterpenes (SQTs), and volatile benzenoids (BZs) (Fig. 1).

In the field, tansy plants with similar chemotypes were not geographically clustered (Fig. S2), indicating that each individual plant was indeed an individual genotype and not a vegetative clone. Previous studies show that minor compounds can also have significant effects on plant-insect interactions^38^,^39^, thus rather than assigning chemotype by the most dominant compounds within the blend, we clustered plants into four classes based on compound concentration^40^. We included all detected compounds rather than limiting the analyses to only the few dominant compounds found in the profile, as has been previously described^27^, to assess the effects of the whole chemical profile rather than giving undue weight to dominant compounds.

The extraction by hexane includes all lipophilic compounds contained within the oil storage cavities and the membranes of the leaf. By contrast, the analysis of headspace from bagged tansy includes all volatile compounds that are emitted from these storage cavities as well compounds that are typically biosynthesised in the tissue prior to being induced due to a variety of biotic and abiotic factors. We hypothesise that it is the compounds that are present both constitutively in the leaf *and* volatile enough to be emitted to the atmosphere (i.e. the compounds ‘likely emitted from storage’) that influence the aphid’s choice in the tansy-aphid system. We thereby only took into account these compounds that are part of the unstressed ‘constitutive’ emission bouquet, which are likely to have an effect on specialists.

When chemotyped according to the terpenes ‘likely emitted from storage’ we found that the chemotypic pattern of the plants had a significant effect on aphid colonisation at the beginning of the season. We disregarded the ‘headspace only compounds’ as they are ubiquitous across plant species and we are looking at aphid colonization of uncolonized or freshly colonized plants, for which the elicited profile may be more variable and less informative.

Some of the constitutively stored compounds are semi-volatile with higher boiling points and low Henry’s law constants (for example borneol, with a boiling point of and 213 °C and a Henry’s law constant of 6.70E-06 Pa m^3^ mol^−1^ 41), and are not volatile enough to be emitted from undisturbed tissue to the atmosphere. Similarly to the compounds found only in the headspace (see Fig. 1), we disregarded these compounds from our analysis as they do not contribute to the distinguishing emission profile. This restriction was statistically justified since when chemotyped using all compounds detected in the hexane fraction we observed no effect of chemotype on any aphid or ant response variable (P > 0.05).

During the main dispersal event (late May/early June) winged aphids may move among host-plants before deciding which plant to settle on and colonise^42^. It is likely that the constitutive emission would be most useful for orientation during this period, particularly since winged aphids have more developed sensory systems than unwinged aphids^43^. If aphids display a particular preference for certain terpene chemotypes then it is reasonable to assume that these chemotypes would be of most benefit to the aphids when they are able to move between plants in search of an acceptable (in terms of nutrient supply, low toxicity, etc.) plant. Early aphid colonisation was positively influenced by the monoterpenes α-thujone and (*E*)-dihydrocarvone, and the sesquiterpenes α-copaene and β-cubebene, and strongly negatively influenced by the monoterpene 4-terpineol. After this main dispersal phase unwinged aphids can still move, but much smaller distances than the winged aphids, and thus new colonisations are more likely on plants close to those already occupied by aphids (Senft *et al*., unpublished data), and then plant chemotype ceases to have an effect on aphid plant selection. Indeed, these aphids have been shown in experimental situations to also preferentially colonise plants already occupied or previously infested with conspecifics, thus strengthening population structuring across different plant chemotypes^44^. Alternatively, host-choice by aphids may be driven by a selective early extinction scenario, whereby aphid colonies can only survive on certain plants (with some chemotypes conferring low growth rate and/or high predation). As we have seen, the overall terpene chemotype also did not have an effect on either of the ant species, whereas individual terpenes could be associated with ant presence. The beneficial ant species *L. niger* was found more often on plants with higher contents of (*Z*)-β-terpineol and α-copaene, and lower (*Z*)-sabinene hydrate and camphene concentrations. While chemotype only had an effect on aphids early in the season (May to June), some individual terpenes explained significant variation in all our variables. This potentially suggests there is even finer-scaled population structuring than the level at which we have analysed here.

Less abundant compounds within an odour profile are rarely investigated^45^, and we did not expect to find that within a plant VOC blend the volatile compounds with the highest effect on aphids were not the most dominant ones. However this is unsurprising as insects have highly sensitive olfactory systems, and non-dominant compounds may act as cues to herbivore enemies^46^.

To determine if the ‘likely emitted from storage’ chemotype is of statistical relevance in other species, we recommend testing the method of chemotyping outlined in this paper using other aromatic plants in future work.

Previous findings by other groups report that one or two compounds/terpenes dominate the odour profile of tansy, with chemotypes being assigned to these compounds. The method of air-drying plant material in order to analyse terpene content, that has been used by other groups^18^,^28^, is not optimal as many volatile compounds are lost during the drying process. Another method that has been used to analyse tansy terpenes is hydro-distillation^24^, however this was also performed on air-dried plant material. While essential oil analysis could give a complex and thorough overview of tansy terpenes it is not practical to measure large numbers of plants. Hexane extraction using freshly frozen harvested material reduces the likelihood that more volatile terpenes are lost during sample preparation. Hexane extraction also allows all lipophilic compounds within glands and membranes to be identified and quantified, thus enabling an analysis with all non-dominant as well as dominant compounds.

To further understand the effect of plant chemotype on the tansy system, it is imperative to have replicate plants, with the same terpene chemotype, that can be used in manipulated experiments. It has been shown in kava (*Piper methysticum* Forst. F.) that variation in kavalactone composition (according to which chemotype was assigned) is very low, and that chemotype remains consistent with clones of the same cultivar^47^. Here we confirm that tansy terpene profiles remain homogenous among daughter clones. This shows that one can split a mother plant into daughter clones that can be used as replicates in future laboratory experiments. Tansy is particularly suitable for this, as it exhibits rhizome growth and therefore we experienced only rare instances of daughter plants not establishing after plants were split.

Overall, we could demonstrate that when chemotyped according to the compounds ‘likely emitted from storage’, the chemical composition of terpenes had an effect on the early colonisation of tansy by aphids during the main dispersal event under field conditions. The differential effect of plant compounds on the two ant species also indicated the potential role of plant chemotype in mediating the interaction between aphids and their mutualistic ants in this metacommunity system. Our work shows that it is not the most dominant compound within a blend that has an effect on aphid colonisation, but rather those less abundant in the plants and thus less often studied in ecological systems. This study furthers our knowledge in the field of chemical ecology by presenting a new method of chemotyping plants, and demonstrating that this new method yields significant results in *Tanacetum vulgare*.

## Materials and Methods

### Study species and system

Tansy (*Tanacetum vulgare* L.) plants were sampled from a field site which is situated north of Freising, Germany. The samplings were performed in July 2013 (for head space analysis) and 2014 (for hexane extraction). All tansy plants were distinguishable from one another and treated as individual ‘islands’. A field survey was conducted in 2014 (Senft *et al*., unpublished data), with every plant visited once per week from May until October collecting data, by counting, on the total number of *Metopeurum fuscoviride* aphids, the presence of *Lasius niger* and *Myrmica rubra*. A plant was considered as colonised by aphids when at least one aphid was observed feeding on the plant, the maximum number of aphids counted in one single week on one plant was 662. Plant location (GPS coordinates) and size at the middle of the season were also recorded.

### Hexane extraction of terpenes and GC-MS analysis

In the 2014 experiment fresh leaves were cut and immediately frozen on dry ice. In the laboratory the frozen leaf samples were ground into a powder and stored at −80 °C until extraction. One ml hexane was added to approximately 500 mg frozen leaf powder, the sample was then vortexed and stored at 4 °C for 24 hours. Amber glass, 1.5 ml screw-top vials with silicone/PTFE septum lids were used to reduce loss of volatiles to the headspace. After 24 hours, 500 μl of the liquid extract was removed and stored at 4 °C. 500 μl hexane was added to the leaf material, which was then vortexed and stored at 4 °C for 24 hours. 500 μl of the liquid extract was again removed and combined with the previously collected extract. One μl of sample was injected into an empty glass cartridge containing a glass micro-vial that was placed on the autosampler of the gas chromatography mass-spectrometer (GC-MS) system. The samples were desorbed from 35 °C to 240 °C at a rate of 120 °C min^−1^ with holding for 2 min in the thermo-desorption unit (TDU, Gerstel, Mülheim an der Ruhr, Germany) coupled to a GC-MS (GC type: 7890A, MS type: 5975C inert XL MSD with a triple axis detector, both from Agilent Technologies (Palo Alto, CA, USA) using a 5% phenyl 95% dimethyl arylene siloxane capillary column (60 m × 250 μm × 0.25 μm DB-5MS + 10 m DG, Agilent Technologies). After initial volatilisation the compounds were refocused on a Tenax liner at −50 °C, then desorbed to 250° at rate of 12 °C min^−1^. Samples were analysed splitlessly at a constant flow rate of He of 1 ml min^−1^ and a temperature program of 40 °C for 0 min, followed by a ramping at 10 °C min^−1^ to 150 °C, then 80 °C min^−1^ to 175 °C, then 5 °C min^−1^ to 190 °C, then 80 °C min^−1^ to 250 °C, then 100 °C min^−1^ to 300 °C and holding for 6 min.

### Headspace collection of VOCs and GC-MS analysis

In the 2013 experiment VOCs were collected from the headspace of plants in the field using the SBSE (stir bar sorptive extraction) method based on non-polar polydimethylsiloxane coated stir bars (Twister, film thickness 0.5 mm, length 10 mm, Gerstel, Mülheim an der Ruhr, Germany).

In the SBSE method VOCs were collected by exposing each twister in the headspace of undamaged leaves enclosed in transparent bags (40 cm length × 31 cm diameter; Toppits^®^ Bratschlauch, Melitta Group, Minden, Germany, PET film, secured at each end) for 3 hours during the day (24.07.2013: sampling time 1000–1600; temperature range 22.7–29.9 °C; Humidity ~60%; no precipitation; from public available data of a weather station at N 48°24′32′, E 11°43′20′ provided by the Bavarian State Research Centre for Agriculture). VOC samples were analysed using the same GC-MS system and column as described above. The temperature program of the GC was set as 40 °C for 2 min, followed by ramping at 6 °C min^−1^ to 80 °C for 3 min, then 3.4 °C min^−1^ to 170 °C, then 12 °C min^−1^ to 300 °C holding for 4 min.

Identification and quantification of all VOCs was performed by comparing the obtained mass spectra with those of commercially available standards (Sigma-Aldrich, Taufkirchen, Germany) or NIST 05 and Wiley library spectra, and the Kovats retention index library^48^.

### Stability of the intrinsic ‘terpene’ chemotype

In 2013 we propagated seven different tansy genotypes in the greenhouse and assessed both the composition and concentration of the compounds present in the spectrum of three clonal plants of each genotype. Leaf hexane extraction and GC-MS analysis were performed as described in the methods above.

### Statistical models

We tested the effect of plant location on plant chemical profile using a Mantel test, correlating matrices of chemical similarity against geographic distance across each pairwise combination of plants in the field. Plants were clustered into four chemotype classes in pvclust() in R^49^ using the Ward.D2 method, correlation distance method and 10000 bootstrap replications. We used generalised linear models with binomial error distribution to test the effect of these chemotype classes on early aphid colonisation during the main dispersal phase with winged aphids present (up to week 25, 16^th^ June) and late aphid colonisation after the main dispersal phase with fewer winged aphids present (after week 25). We also used the same method to test for the effect of chemotype classes on the presence of aphid-tending ants (*L. niger* and *M. rubra*) before aphids colonised the plant, in order to disentangle effects of aphid-tending. For this we used the cbind() function in R to combine the number of times ants were present on the plant before aphid colonisation, with the total number of observations. This accounts for variation among aphid colonisation dates.

To test the influence of plant emitted compounds on the associated invertebrate community we used Bayesian Model Averaging (BMA) using the function bic.glm (for binomial models, presence absence data) as implemented in the R package BMA^50^. BMA provides a method through which to account for correlation among variables and the uncertainty associated with model choice through order effects, and is preferable compared to other methods used such as stepwise regression parameters as it accounts for uncertainty in both model selection and parameter estimates simultaneously. In BMA, multiple models are run and only those retained have posterior probabilities (that the coefficient for each predictor does not equal 0) within a factor of 1/20 of that of the best model (Occam’s window), following the methods used in ref. 51. For each explanatory variable, the posterior effect probability (PEP) is then equivalent to the proportion of models in which the variable was retained. When two chemicals were correlated, only one would be retained in any model. We used the information from the top five models to infer those compounds that are explaining the greatest amount of variation among our response variables. Our response variables were the same as used to test for the effect of the chemotype classes (i.e. early aphid colonisation, late aphid colonisation, presence of *L. niger* and *M. rubra* before aphid colonisation). Additional covariates used in all models were plant size and plant accessibility. The accessibility is defined as the proportion of a plant that is not surrounded by other tansy plants and ranges from 0–360 degrees; a plant with an accessibility of 0 is completely surrounded by tansy plants (low accessibility) and a plant with the accessibility of 360 is not surrounded at all (high accessibility) (Senft *et al*., unpublished data)). For the aphid models, we also included a covariate of ant presence before aphid colonisation. We assessed the robustness of this analysis method by correlating the total amount of influence of each chemical variables (sum of PEP values) firstly to the total concentration of the chemical (to test for the influence of dominance) and then also to the variance in chemical concentrations (to test for the influence of highly variable chemicals). These analyses were all run using R v.3.2.0^52^ and RStudio v.0.98.977^53^.

## Additional Information

**How to cite this article**: Clancy, M. V. *et al*. Chemotypic variation in terpenes emitted from storage pools influences early aphid colonisation on tansy. *Sci. Rep.* **6**, 38087; doi: 10.1038/srep38087 (2016).

**Publisher's note:** Springer Nature remains neutral with regard to jurisdictional claims in published maps and institutional affiliations.

## Supplementary Material

Supplementary Data

Supplementary Dataset
