# Supplementary material for: Chemotypic variation in terpenes emitted from storage pools influences early aphid colonisation on tansy
Source: Sci Rep. 2016 Nov 28;6:38087. doi: 10.1038/srep38087 (PMC5125103; doi:10.1038/srep38087)

**Chemotypic variation in terpenes emitted from storage pools influences early aphid colonisation on tansy**

Mary V. Clancy^a^, Sharon E. Zytynska^b^, Matthias Senft^b^, Wolfgang W. Weisser^b^ and Jörg-Peter Schnitzler^a*^

^a^ *Helmholtz Zentrum München, Department of Environmental Sciences (DES), Research Unit Environmental Simulation (EUS), Ingolstädter Landstr. 1, 85764 Neuherberg, Germany*

^b^ *Technische Universität München, Terrestrial Ecology Research Group, Department of Ecology and Ecosystem Management, School of Life Sciences Weihenstephan, Hans-Carl-von-Carlowitz-Platz 2, 85354 Freising, Germany*

^*^ Corresponding author: Jörg-Peter Schnitzler; +498931872413, Fax +498931874431,
jp.schnitzler@helmholtz-muenchen.de

**Supplementary tables**

**Table S1.** Concentrations of all compounds identified in leaf hexane extracts of 176 plants at field site.

**Table S2.** Summary of the Bayesian Model Averaging (BMA) Posterior Effect Probabilities scores across all variables by compound (sumPEP) and the mean concentration and variation (using standard deviation scores) of the concentrations across all plants.

**Table S3a.** Effect of concentration of putatively emitted compounds on aphid early colonisation

**Table S3b.** Effect of concentration of putatively emitted compounds on aphid late colonisation

**Table S3c.** Effect of concentration of putatively emitted compounds on the presence of *Lasius niger* ants before aphid colonisation

**Table S3d.** Effect of concentration of putatively emitted compounds on the presence of *Myrmia rubra* ants before aphid colonisation

**Table S4a.** Effect of concentration of stored compounds on aphid early colonisation

**Table S4b.** Effect of concentration of stored compounds on aphid late colonisation

**Table S4c.** Effect of concentration of stored compounds on the presence of *Lasius niger* ants before aphid colonisation

**Table S4d.** Effect of concentration of stored compounds on the presence of *Myrmica rubra* ants before aphid colonisation

**Supplementary figures**

**Figure S1:** Full correlation matrix of all putatively emitted compounds

**Figure S2:** Map of the field site showing chemotypes

**Table S1.** Concentrations of all compounds identified in leaf hexane extracts of 176 plants at field site.

Table in separate Excel file.

**Table S2. Summary of the Bayesian Model Averaging (BMA) Posterior Effect Probabilities scores across all variables by compound (sumPEP) and the mean concentration and variation (using standard deviation scores) of the concentrations across all plants. To show that those compounds with high PEP (high influence) are not those with the most concentration or the most variation across plants**

| **Compound** | ***sumPEP*** | **Mean compound concentration** | **Standard deviation of compound concentration** |
| --- | --- | --- | --- |
| ***Monoterpenes*** |  |  |  |
| α-Thujene | 31.9 | 0.0200 | 0.0276 |
| α-Pinene | 5.0 | 0.0067 | 0.0129 |
| Camphene | 62.9 | 0.0075 | 0.0116 |
| Sabinene | 23.2 | 0.0597 | 0.0519 |
| α-Terpinene | 104.5 | 0.0038 | 0.0055 |
| o-Cymene | 13.7 | 0.0120 | 0.0130 |
| Limonene | 12.6 | 0.0005 | 0.0011 |
| γ-Terpinene | 110.5 | 0.0132 | 0.0162 |
| α-Thujone | 34.8 | 0.1514 | 0.3118 |
| ***Oxygenated Monoterpenes*** |  |  |  |
| Eucaliptol | 13.1 | 0.1510 | 0.2106 |
| (*Z*)-β-Terpineol | 70.2 | 0.0794 | 0.1163 |
| (*Z*)-Sabinene Hydrate | 89.5 | 0.0146 | 0.0157 |
| L-Camphor | 61.4 | 0.1872 | 0.3886 |
| 4-Terpineol | 176.4 | 0.0072 | 0.0078 |
| Terpineol | 11.6 | 0.0018 | 0.0035 |
| (*E*)-Dihydrocarvone | 40.2 | 0.0009 | 0.0009 |
| ***Sesquiterpenes*** |  |  |  |
| α-Copaene | 56.3 | 0.0022 | 0.0018 |
| (*E*)-β-Caryophyllene | 20.5 | 0.0007 | 0.0035 |
| β-Cubebene | 38.3 | 0.0590 | 0.0457 |
| α-Amorphene | 26.4 | 0.0034 | 0.0017 |
| Germacrene D | 19.2 | 0.0016 | 0.0012 |
| β-Sesquiphellandrene | 9.5 | 0.0008 | 0.0016 |

**Table S3a. Effect of concentration of putatively emitted compounds on aphid early colonisation**

| **Response:** | **Aphid Early Colonisation (binomial)** | | | | | | | |
| --- | --- | --- | --- | --- | --- | --- | --- | --- |
|  |  |  |  | **Five best models** | | | | |
| **Predictors** | **PEP** | **EV** | **SD** | **Model 1** | **Model 2** | **Model 3** | **Model 4** | **Model 5** |
| Intercept | 100 | -2.69 | 0.67 | -2.308 | -2.742 | -2.775 | -2.724 | -2.661 |
| ***Covariates*** |  |  |  |  |  |  |  |  |
| *Lasius niger* | 2.3 | -0.02 | 0.15 |  |  |  |  |  |
| *Myrmia rubra* | 1.4 | 0.00 | 0.08 |  |  |  |  |  |
| Plant size | 93.4 | 1.53 | 0.72 | 1.522 | 1.720 | 1.668 | 1.620 | 1.566 |
| Accessibility | 100 | 0.01 | 0.00 | 0.014 | 0.013 | 0.014 | 0.014 | 0.014 |
| ***Monoterpenes*** |  |  |  |  |  |  |  |  |
| α-Thujene | 5.9 | -1.69 | 8.33 |  |  |  |  |  |
| α-Pinene | 1.4 | -0.17 | 3.32 |  |  |  |  |  |
| Camphene | 2.1 | 0.23 | 3.49 |  |  |  |  |  |
| Sabinene | 8.3 | 0.67 | 2.87 |  |  |  |  |  |
| α-Terpinene | 2.4 | 1.21 | 11.99 |  |  |  |  |  |
| o-Cymene | 4.2 | 0.83 | 5.50 |  |  |  |  |  |
| Limonene | 8.4 | 20.74 | 85.42 |  |  |  |  |  |
| γ-Terpinene | 2.5 | 0.48 | 4.52 |  |  |  |  |  |
| α-Thujone | 20.2 | 0.24 | 0.55 |  |  |  |  | 1.057 |
| ***Oxygenated Monoterpenes*** | | |  |  |  |  |  |  |
| Eucaliptol | 1.3 | 0.00 | 0.13 |  |  |  |  |  |
| (*Z*)-β-Terpineol | 2.0 | 0.04 | 0.51 |  |  |  |  |  |
| (*Z*)-Sabinene Hydrate | 11.5 | -5.79 | 18.77 |  |  |  |  |  |
| L-Camphor | 2.1 | 0.01 | 0.10 |  |  |  |  |  |
| 4-Terpineol | 82.6 | -94.93 | 58.44 | -108.015 | -122.775 | -115.590 | -118.875 | -86.675 |
| Terpineol | 1.3 | -0.22 | 6.75 |  |  |  |  |  |
| (*E*)-Dihydrocarvone | 21.9 | 99.39 | 218.60 |  |  | 455.516 |  |  |
| ***Sesquiterpenes*** |  |  |  |  |  |  |  |  |
| α-Copaene | 13.6 | 26.18 | 77.60 |  |  |  | 201.677 |  |
| (*E*)-β-Caryophyllene | 4.7 | 2.86 | 17.54 |  |  |  |  |  |
| β-Cubebene | 22.7 | 1.93 | 4.16 |  | 8.689 |  |  |  |
| α-Amorphene | 7.5 | 13.43 | 63.41 |  |  |  |  |  |
| Germacrene D | 3.2 | -5.86 | 45.64 |  |  |  |  |  |
| β-Sesquiphellandrene | 1.5 | 1.00 | 15.44 |  |  |  |  |  |
| **BIC** |  |  |  | -692.4 | -692.1 | -691.6 | -691.2 | -690.6 |
| **Posterior Probability** |  |  |  | 0.062 | 0.054 | 0.042 | 0.035 | 0.026 |

Bayesian Model Averaging (BMA package in R) used to assess effect of the concentration of each individual compound

**Table S3b. Effect of concentration of putatively emitted compounds on aphid late colonisation**

| **Response:** | **Aphid Late Colonisation (binomial)** | | | | | | | | | | | | | | | |  |
| --- | --- | --- | --- | --- | --- | --- | --- | --- | --- | --- | --- | --- | --- | --- | --- | --- | --- |
|  |  | |  | |  | | **Five best models** | | | | | | | | | |  |
| **Predictors** | **PEP** | | **EV** | | **SD** | | **Model 1** | | **Model 2** | | **Model 3** | | **Model 4** | | **Model 5** | |  |
| Intercept | 100 | | -0.21 | | 0.54 | | -0.380 | | 0.011 | | -0.338 | | -0.699 | | -0.724 | |  |
| ***Covariates*** | |  | |  | |  | |  | |  | |  | |  | |  | |
| *Lasius niger* | 100 | | 2.47 | | 0.75 | | 2.545 | | 2.230 | | 2.664 | | 2.501 | | 2.493 | |  |
| *Myrmia rubra* | 57.1 | | 0.85 | | 0.88 | | 1.464 | |  | | 1.557 | | 1.477 | | 1.554 | |  |
| Plant size | 1.5 | | -0.01 | | 0.10 | |  | |  | |  | |  | |  | |  |
| Accessibility | 100 | | -0.01 | | 0.00 | | -0.013 | | -0.012 | | -0.012 | | -0.012 | | -0.013 | |  |
| ***Monoterpenes*** | |  | |  | |  | |  | |  | |  | |  | |  | |
| α-Thujene | 24.3 | | 5.81 | | 13.06 | |  | |  | | 34.736 | | 10.724 | |  | |  |
| α-Pinene | 1.4 | | -0.13 | | 2.17 | |  | |  | |  | |  | |  | |  |
| Camphene | 1.6 | | -0.22 | | 2.68 | |  | |  | |  | |  | |  | |  |
| Sabinene | 12.8 | | -1.83 | | 5.65 | |  | |  | | -16.294 | |  | |  | |  |
| α-Terpinene | 5.6 | | 2.82 | | 14.14 | |  | |  | |  | |  | |  | |  |
| o-Cymene | 5.3 | | -1.13 | | 5.99 | |  | |  | |  | |  | |  | |  |
| Limonene | 1.2 | | 0.77 | | 18.18 | |  | |  | |  | |  | |  | |  |
| γ-Terpinene | 11.2 | | 2.36 | | 8.04 | |  | |  | |  | |  | | 17.687 | |  |
| α-Thujone | 1.3 | | 0.00 | | 0.08 | |  | |  | |  | |  | |  | |  |
| ***Oxygenated Monoterpenes*** | | | | | |  | |  | |  | |  | |  | |  | |
| Eucaliptol | 3.6 | | -0.04 | | 0.30 | |  | |  | |  | |  | |  | |  |
| (*Z*)-β-Terpineol | 4.1 | | -0.10 | | 0.72 | |  | |  | |  | |  | |  | |  |
| (*Z*)-Sabinene Hydrate | 4.6 | | -0.90 | | 5.72 | |  | |  | |  | |  | |  | |  |
| L-Camphor | 1.8 | | -0.01 | | 0.08 | |  | |  | |  | |  | |  | |  |
| 4-Terpineol | 1.6 | | 0.33 | | 3.91 | |  | |  | |  | |  | |  | |  |
| Terpineol | 6.8 | | -6.65 | | 31.64 | |  | |  | |  | |  | |  | |  |
| (*E*)-Dihydrocarvone | 11.5 | | -44.15 | | 147.40 | |  | |  | |  | |  | |  | |  |
| ***Sesquiterpenes*** | |  | |  | |  | |  | |  | |  | |  | |  | |
| α-Copaene | 1.7 | | -1.52 | | 17.28 | |  | |  | |  | |  | |  | |  |
| (*E*)-β-Caryophyllene | 3.8 | | -2.62 | | 18.64 | |  | |  | |  | |  | |  | |  |
| β-Cubebene | 6.8 | | -0.54 | | 2.47 | |  | |  | |  | |  | |  | |  |
| α-Amorphene | 3 | | -5.53 | | 43.18 | |  | |  | |  | |  | |  | |  |
| Germacrene D | 11.4 | | 32.37 | | 108.40 | |  | |  | |  | |  | |  | |  |
| β-Sesquiphellandrene | 1.2 | | -0.40 | | 12.30 | |  | |  | |  | |  | |  | |  |
| **BIC** |  | |  | |  | | -680.5 | | -680.0 | | -679.6 | | -679.6 | | -679.5 | |  |
| **Posterior Probability** |  | |  | |  | | 0.046 | | 0.035 | | 0.03 | | 0.029 | | 0.027 | |  |

Bayesian Model Averaging (BMA package in R) used to assess effect of the concentration of each individual compound

**Table S3c. Effect of concentration of putatively emitted compounds on the presence of *Lasius niger* ants before aphid colonisation**

| **Response:** | **Presence of *Lasius niger* ants before aphid colonisation** | | | | | | | | | | | | | | |  |
| --- | --- | --- | --- | --- | --- | --- | --- | --- | --- | --- | --- | --- | --- | --- | --- | --- |
|  |  | |  | |  | **Five best models** | | | | | | | | | |  |
| **Predictors** | **PEP** | | **EV** | | **SD** | **Model 1** | | **Model 2** | | **Model 3** | | **Model 4** | | **Model 5** | |  |
| Intercept | 100 | | -1.32 | | 0.26 | -1.106 | | -1.212 | | -1.090 | | -1.435 | | -0.964 | |  |
| ***Covariates*** |  |  | |  | | |  | |  | |  | |  | |  | |
| Plant size | 30.1 | | 0.12 | | 0.20 |  | | 0.411 | |  | |  | |  | |  |
| Accessibility | 64.3 | | 0.00 | | 0.00 | -0.003 | | -0.003 | | -0.003 | |  | | -0.003 | |  |
| ***Monoterpenes*** |  |  | |  | | |  | |  | |  | |  | |  | |
| α-Thujene | 0.2 | | 0.00 | | 0.14 |  | |  | |  | |  | |  | |  |
| α-Pinene | 0.8 | | 0.02 | | 0.39 |  | |  | |  | |  | |  | |  |
| Camphene | 57.6 | | -28.12 | | 27.17 | -51.740 | | -51.630 | |  | | -52.420 | | -46.830 | |  |
| Sabinene | 0.2 | | 0.00 | | 0.07 |  | |  | |  | |  | |  | |  |
| α-Terpinene | 0.2 | | 0.00 | | 0.68 |  | |  | |  | |  | |  | |  |
| o-Cymene | 1.1 | | -0.05 | | 0.71 |  | |  | |  | |  | |  | |  |
| Limonene | 1.1 | | -0.91 | | 13.18 |  | |  | |  | |  | |  | |  |
| γ-Terpinene | 0.5 | | 0.02 | | 0.42 |  | |  | |  | |  | |  | |  |
| α-Thujone | 1.0 | | 0.00 | | 0.03 |  | |  | |  | |  | |  | |  |
| ***Oxygenated Monoterpenes*** | | | |  | | |  | |  | |  | |  | |  | |
| Eucaliptol | 6.6 | | -0.04 | | 0.16 |  | |  | |  | |  | |  | |  |
| (*Z*)-β-Terpineol | 62.7 | | 1.83 | | 1.58 | 2.952 | | 3.015 | | 2.957 | | 2.639 | | 3.056 | |  |
| (*Z*)-Sabinene Hydrate | 67.8 | | -16.80 | | 13.73 | -28.230 | | -28.640 | | -24.300 | | -24.210 | | -27.080 | |  |
| L-Camphor | 56.0 | | 0.68 | | 0.66 | 1.208 | | 1.170 | |  | | 1.289 | | 1.146 | |  |
| 4-Terpineol | 4.1 | | 0.43 | | 4.28 |  | |  | |  | |  | |  | |  |
| Terpineol | 1.8 | | -0.35 | | 3.57 |  | |  | |  | |  | |  | |  |
| (*E*)-Dihydrocarvone | 4.7 | | 0.86 | | 35.70 |  | |  | |  | |  | |  | |  |
| ***Sesquiterpenes*** |  |  | |  | | |  | |  | |  | |  | |  | |
| α-Copaene | 39.3 | | 32.06 | | 45.94 | 83.230 | | 85.300 | |  | | 85.020 | |  | |  |
| (*E*)-β-Caryophyllene | 1.0 | | 0.14 | | 2.32 |  | |  | |  | |  | |  | |  |
| β-Cubebene | 7.0 | | 0.19 | | 0.82 |  | |  | |  | |  | |  | |  |
| α-Amorphene | 12.7 | | 10.44 | | 30.98 |  | |  | |  | |  | |  | |  |
| Germacrene D | 3.2 | | 2.43 | | 16.43 |  | |  | |  | |  | |  | |  |
| β-Sesquiphellandrene | 5.4 | | 3.04 | | 15.61 |  | |  | |  | |  | |  | |  |
| **BIC** |  | |  | |  | -721.7 | | -721.2 | | -721.0 | | -721.0 | | -720.8 | |  |
| **Posterior Probability** |  | |  | |  | 0.045 | | 0.035 | | 0.032 | | 0.031 | | 0.029 | |  |

Bayesian Model Averaging (BMA package in R) used to assess effect of the concentration of each individual compound

**Table S3d Effect of concentration of putatively emitted compounds on the presence of *Myrmia rubra* ants before aphid colonisation**

| **Response:** | **Presence of *Myrmia rubra* ants before aphid colonisation (binomial)** | | | | | | | | | | | | | | | | |
| --- | --- | --- | --- | --- | --- | --- | --- | --- | --- | --- | --- | --- | --- | --- | --- | --- | --- |
|  |  | |  | | |  | | **Five best models** | | | | | | | | | |
| **Predictors** | **PEP** | | **EV** | | | **SD** | | **Model 1** | | **Model 2** | | **Model 3** | | **Model 4** | | **Model 5** | |
| Intercept | 100 | | -1.45 | | | 0.11 | | -1.411 | | -1.494 | | -1.441 | | -1.527 | | -1.491 | |
| ***Covariates*** | |  | |  |  | |  | |  | |  | |  | |  | |  |
| Plant size | 8.8 | | 0.03 | | | 0.10 | |  | |  | |  | | 0.299 | |  | |
| Accessibility | 3.4 | | 0.00 | | | 0.00 | |  | |  | |  | |  | |  | |
| ***Monoterpenes*** | |  | |  |  | |  | |  | |  | |  | |  | |  |
| α-Thujene | 1.5 | | -0.02 | | | 0.63 | |  | |  | |  | |  | |  | |
| α-Pinene | 1.4 | | 0.00 | | | 0.40 | |  | |  | |  | |  | |  | |
| Camphene | 1.6 | | 0.04 | | | 0.75 | |  | |  | |  | |  | |  | |
| Sabinene | 1.9 | | -0.02 | | | 0.29 | |  | |  | |  | |  | |  | |
| α-Terpinene | 96.3 | | -187.60 | | | 62.69 | | -194.268 | | -197.135 | | -203.826 | | -194.229 | | -181.611 | |
| o-Cymene | 3.1 | | 0.16 | | | 1.13 | |  | |  | |  | |  | |  | |
| Limonene | 1.9 | | 1.05 | | | 12.69 | |  | |  | |  | |  | |  | |
| γ-Terpinene | 96.3 | | 66.48 | | | 21.37 | | 70.214 | | 72.178 | | 71.868 | | 70.491 | | 52.735 | |
| α-Thujone | 12.3 | | 0.05 | | | 0.17 | |  | | 0.426 | |  | |  | |  | |
| ***Oxygenated Monoterpenes*** | | | | |  | |  | |  | |  | |  | |  | |  |
| Eucaliptol | 1.6 | | 0.00 | | | 0.04 | |  | |  | |  | |  | |  | |
| (*Z*)-β-Terpineol | 1.4 | | 0.00 | | | 0.08 | |  | |  | |  | |  | |  | |
| (*Z*)-Sabinene Hydrate | 5.6 | | 0.63 | | | 3.09 | |  | |  | |  | |  | |  | |
| L-Camphor | 1.5 | | 0.00 | | | 0.02 | |  | |  | |  | |  | |  | |
| 4-Terpineol | 88.1 | | -30.12 | | | 15.88 | | -33.799 | | -31.549 | | -31.482 | | -33.891 | |  | |
| Terpineol | 1.7 | | -0.15 | | | 2.19 | |  | |  | |  | |  | |  | |
| (*E*)-Dihydrocarvone | 2.1 | | 1.38 | | | 14.31 | |  | |  | |  | |  | |  | |
| ***Sesquiterpenes*** | |  | |  |  | |  | |  | |  | |  | |  | |  |
| α-Copaene | 1.7 | | 0.37 | | | 5.41 | |  | |  | |  | |  | |  | |
| (*E*)-β-Caryophyllene | 11.0 | | 4.01 | | | 12.97 | |  | |  | | 34.816 | |  | |  | |
| β-Cubebene | 1.8 | | -0.02 | | | 0.24 | |  | |  | |  | |  | |  | |
| α-Amorphene | 3.2 | | -1.54 | | | 10.92 | |  | |  | |  | |  | |  | |
| Germacrene D | 1.4 | | -0.12 | | | 6.18 | |  | |  | |  | |  | |  | |
| β-Sesquiphellandrene | 1.4 | | 0.05 | | | 4.62 | |  | |  | |  | |  | |  | |
| **BIC** |  | |  | | |  | | -679.7 | | -677.3 | | -677.0 | | -676.6 | | -676.2 | |
| **Posterior Probability** |  | |  | | |  | | 0.263 | | 0.079 | | 0.07 | | 0.057 | | 0.047 | |

Bayesian Model Averaging (BMA package in R) used to assess effect of the concentration of each individual compound

**Table S4a. Effect of concentration of stored compounds on aphid early colonisation**

| **Response:** | **Aphid Early Colonisation (binomial)** | | | | | | | | | | | | | | | |
| --- | --- | --- | --- | --- | --- | --- | --- | --- | --- | --- | --- | --- | --- | --- | --- | --- |
|  |  | |  | |  | | | **Five best models** | | | | | | | | |
| **Predictors** | **PEP** | | **EV** | | **SD** | | | **Model 1** | | **Model 2** | | **Model 3** | | **Model 4** | | **Model 5** |
| Intercept | 100 | | -3.13 | | 0.66 | | | -2.750 | | -2.997 | | -2.846 | | -3.442 | | -3.383 |
| ***Covariates*** | |  | |  | |  |  | |  | |  | |  | |  | |
| *Lasius niger* | 3 | | -0.02 | | 0.17 | | |  | |  | |  | |  | |  |
| *Myrmia rubra* | 2.6 | | 0.01 | | 0.13 | | |  | |  | |  | |  | |  |
| Plant size | 98.3 | | 1.80 | | 0.68 | | | 1.782 | | 1.648 | | 1.806 | | 1.934 | | 1.927 |
| Accessibility | 100 | | 0.01 | | 0.00 | | | 0.014 | | 0.014 | | 0.013 | | 0.014 | | 0.014 |
| ***Monoterpenes*** | |  | |  | |  |  | |  | |  | |  | |  | |
| β-Pinene | 7.3 | | -7.23 | | 32.74 | | |  | |  | |  | |  | |  |
| ***Monoterpene Acetates*** | | | | | |  |  | |  | |  | |  | |  | |
| Verbenyl Acetate | 1.3 | | -0.11 | | 3.16 | | |  | |  | |  | |  | |  |
| Bornyl Acetate | 1.9 | | 0.23 | | 2.74 | | |  | |  | |  | |  | |  |
| Dihydrocarvyl Acetate | 1.8 | | -0.01 | | 0.20 | | |  | |  | |  | |  | |  |
| Isopulegol Acetate | 1.6 | | 0.00 | | 0.07 | | |  | |  | |  | |  | |  |
| Myrtenyl Acetate | 1 | | -0.01 | | 0.68 | | |  | |  | |  | |  | |  |
| α-Terpinyl Acetate | 70.5 | | -72.99 | | 61.82 | | | -92.558 | |  | | -112.599 | | -101.251 | | -98.076 |
| ***Oxygenated Monoterpenes*** | | | | | |  |  | |  | |  | |  | |  | |
| Sabinol | 1 | | 0.04 | | 17.07 | | |  | |  | |  | |  | |  |
| Berbenol | 3.2 | | 1.35 | | 16.81 | | |  | |  | |  | |  | |  |
| Camphenol-6 | 1 | | 0.11 | | 3.45 | | |  | |  | |  | |  | |  |
| Borneol | 1.9 | | -0.16 | | 2.58 | | |  | |  | |  | |  | |  |
| Myrtenol | 7.8 | | -2.56 | | 11.44 | | |  | |  | |  | |  | |  |
| (*Z*)-Carveol | 1.7 | | -0.06 | | 0.82 | | |  | |  | |  | |  | |  |
| ***Sesquiterpenes*** | |  | |  | |  |  | |  | |  | |  | |  | |
| γ-Elemene | 5 | | -15.27 | | 95.21 | | |  | |  | |  | |  | |  |
| Sesquiterpene #1 | 11.2 | | 0.60 | | 1.93 | | |  | |  | |  | |  | |  |
| Sesquiterpene #2 | 13.6 | | 1.73 | | 5.00 | | |  | |  | |  | |  | | 12.858 |
| Sesquiterpene #3 | 9.4 | | 4.06 | | 17.30 | | |  | |  | |  | |  | |  |
| γ-Muurolene | 5.5 | | 1.28 | | 9.68 | | |  | |  | |  | |  | |  |
| δ-Cadenine | 1.7 | | 0.01 | | 1.42 | | |  | |  | |  | |  | |  |
| Germacrene B | 15.5 | | 6.61 | | 18.37 | | |  | |  | |  | | 42.030 | |  |
| Isolongifolene | 2.7 | | -3.04 | | 40.67 | | |  | |  | |  | |  | |  |
| Cedrene-13-ol, 8 | 1.3 | | 0.82 | | 28.21 | | |  | |  | |  | |  | |  |
| α-Cadinol | 21.4 | | 77.00 | | 238.40 | | |  | |  | | 289.535 | |  | |  |
| allo-Aromadendrene | 11 | | 10.01 | | 383.60 | | |  | |  | |  | |  | |  |
| **BIC** |  | |  | |  | | | -686.0 | | -685.5 | | -685.3 | | -685.1 | | -684.9 |
| **Posterior Probability** |  | |  | |  | | | 0.049 | | 0.039 | | 0.036 | | 0.031 | | 0.029 |

Bayesian Model Averaging (BMA package in R) used to assess effect of the concentration of each individual compound

**Table S4b. Effect of concentration of stored compounds on aphid late colonisation**

| **Response:** | **Aphid Late Colonisation (binomial)** | | | | | | | | | | | | | | | | |
| --- | --- | --- | --- | --- | --- | --- | --- | --- | --- | --- | --- | --- | --- | --- | --- | --- | --- |
|  |  | |  | | |  | | **Five best models** | | | | | | | | | |
| **Predictors** | **PEP** | | **EV** | | | **SD** | | **Model 1** | | **Model 2** | | **Model 3** | | **Model 4** | | **Model 5** | |
| Intercept | 100 | | 0.26 | | | 0.63 | | -0.436 | | 0.166 | | 0.236 | | 0.575 | | -0.029 | |
| ***Covariates*** | |  | |  |  | |  | |  | |  | |  | |  | |  |
| *Lasius niger* | 100 | | 2.76 | | | 0.80 | | 2.645 | | 3.029 | | 3.032 | | 2.676 | | 2.311 | |
| *Myrmia rubra* | 61.2 | | 0.95 | | | 0.91 | | 1.516 | | 1.492 | | 1.525 | |  | |  | |
| Plant size | 2.8 | | -0.02 | | | 0.16 | |  | |  | |  | |  | |  | |
| Accessibility | 100 | | -0.01 | | | 0.00 | | -0.014 | | -0.015 | | -0.014 | | -0.013 | | -0.012 | |
| ***Monoterpenes*** | |  | |  |  | |  | |  | |  | |  | |  | |  |
| β-Pinene | 1.3 | | -0.23 | | | 6.25 | |  | |  | |  | |  | |  | |
| ***Monoterpene Acetates*** | | | | |  | |  | |  | |  | |  | |  | |  |
| Verbenyl Acetate | 1.8 | | -0.42 | | | 5.46 | |  | |  | |  | |  | |  | |
| Bornyl Acetate | 1.3 | | 0.03 | | | 1.92 | |  | |  | |  | |  | |  | |
| Dihydrocarvyl Acetate | 3.0 | | -0.02 | | | 0.21 | |  | |  | |  | |  | |  | |
| Isopulegol Acetate | 1.0 | | 0.00 | | | 0.06 | |  | |  | |  | |  | |  | |
| Myrtenyl Acetate | 2.2 | | -0.10 | | | 1.09 | |  | |  | |  | |  | |  | |
| α-Terpinyl Acetate | 4.2 | | 1.42 | | | 9.19 | |  | |  | |  | |  | |  | |
| ***Oxygenated Monoterpenes*** | | | | |  | |  | |  | |  | |  | |  | |  |
| Sabinol | 1.8 | | 1.96 | | | 27.08 | |  | |  | |  | |  | |  | |
| Berbenol | 24.5 | | -232.73 | | | 494.20 | |  | |  | |  | |  | |  | |
| Camphenol-6 | 4.6 | | -2.07 | | | 12.06 | |  | |  | |  | |  | |  | |
| Borneol | 2.1 | | -0.22 | | | 2.68 | |  | |  | |  | |  | |  | |
| Myrtenol | 2.6 | | -0.33 | | | 3.16 | |  | |  | |  | |  | |  | |
| (*Z*)-Carveol | 3.2 | | -0.13 | | | 1.04 | |  | |  | |  | |  | |  | |
| ***Sesquiterpenes*** | |  | |  |  | |  | |  | |  | |  | |  | |  |
| γ-Elemene | 4.9 | | 12.68 | | | 74.80 | |  | |  | |  | |  | |  | |
| Sesquiterpene #1 | 27.6 | | -2.49 | | | 5.98 | |  | | -7.630 | |  | | -7.817 | |  | |
| Sesquiterpene #2 | 22.2 | | -3.58 | | | 12.77 | |  | |  | | -16.886 | |  | |  | |
| Sesquiterpene #3 | 8.5 | | -3.81 | | | 34.83 | |  | |  | |  | |  | |  | |
| γ-Muurolene | 5.9 | | 2.65 | | | 16.88 | |  | |  | |  | |  | |  | |
| δ-Cadenine | 2.3 | | -0.15 | | | 1.81 | |  | |  | |  | |  | |  | |
| Germacrene B | 14.1 | | -6.57 | | | 24.98 | |  | |  | |  | |  | |  | |
| Isolongifolene | 4.7 | | -14.06 | | | 87.77 | |  | |  | |  | |  | |  | |
| Cedrene-13-ol, 8 | 14 | | -72.41 | | | 215.70 | |  | |  | |  | |  | |  | |
| α-Cadinol | 3.2 | | -4.44 | | | 34.33 | |  | |  | |  | |  | |  | |
| allo-Aromadendrene | 2.0 | | -3.57 | | | 42.45 | |  | |  | |  | |  | |  | |
| **BIC** |  | |  | | |  | | -678.0 | | -677.7 | | -677.5 | | -677.4 | | -677.2 | |
| **Posterior Probability** |  | |  | | |  | | 0.032 | | 0.028 | | 0.026 | | 0.025 | | 0.023 | |

Bayesian Model Averaging (BMA package in R) used to assess effect of the concentration of each individual compound

**Table S4c. Effect of concentration of stored compounds on the presence of *Lasius niger* ants before aphid colonisation**

| **Response:** | **Presence of *Lasius niger* ants before aphid colonisation (binomial)** | | | | | | | | | | | | | | | | |
| --- | --- | --- | --- | --- | --- | --- | --- | --- | --- | --- | --- | --- | --- | --- | --- | --- | --- |
|  |  | |  | | |  | | **Five best models** | | | | | | | | | |
| **Predictors** | **PEP** | | **EV** | | | **SD** | | **Model 1** | | **Model 2** | | **Model 3** | | **Model 4** | | **Model 5** | |
| Intercept | 100 | | -1.62 | | | 0.18 | | -1.716 | | -1.700 | | -1.691 | | -1.667 | | -1.472 | |
| ***Covariates*** | |  | |  |  | |  | |  | |  | |  | |  | |  |
| Plant size | 11.1 | | 0.04 | | | 0.12 | |  | |  | |  | |  | |  | |
| Accessibility | 15.3 | | 0.00 | | | 0.00 | |  | |  | |  | |  | |  | |
| ***Monoterpenes*** | |  | |  |  | |  | |  | |  | |  | |  | |  |
| β-Pinene | 1.5 | | -0.60 | | | 5.73 | |  | |  | |  | |  | |  | |
| ***Monoterpene Acetates*** | | | | |  | |  | |  | |  | |  | |  | |  |
| Verbenyl Acetate | 60.1 | | -15.82 | | | 15.32 | | -27.841 | | -26.159 | | -28.348 | | -27.285 | |  | |
| Bornyl Acetate | 0.5 | | 0.04 | | | 0.75 | |  | |  | |  | |  | |  | |
| Dihydrocarvyl Acetate | 9.3 | | 0.02 | | | 0.09 | |  | |  | |  | |  | |  | |
| Isopulegol Acetate | 65.2 | | 0.20 | | | 0.17 | | 0.305 | | 0.312 | |  | | 0.302 | | 0.292 | |
| Myrtenyl Acetate | 4.6 | | 0.12 | | | 0.64 | |  | |  | |  | |  | |  | |
| α-Terpinyl Acetate | 4.8 | | -0.88 | | | 4.61 | |  | |  | |  | |  | |  | |
| ***Oxygenated Monoterpenes*** | | | | |  | |  | |  | |  | |  | |  | |  |
| Sabinol | 0.1 | | 0.01 | | | 2.21 | |  | |  | |  | |  | |  | |
| Berbenol | 39.7 | | -133.10 | | | 191.70 | |  | |  | |  | |  | | -332.436 | |
| Camphenol-6 | 1.3 | | 0.23 | | | 2.43 | |  | |  | |  | |  | |  | |
| Borneol | 4.0 | | 0.41 | | | 2.35 | |  | |  | |  | |  | |  | |
| Myrtenol | 0.1 | | 0.00 | | | 0.15 | |  | |  | |  | |  | |  | |
| (*Z*)-Carveol | 4.1 | | -0.09 | | | 0.54 | |  | |  | |  | |  | |  | |
| ***Sesquiterpenes*** | |  | |  |  | |  | |  | |  | |  | |  | |  |
| γ-Elemene | 0.5 | | -0.21 | | | 8.69 | |  | |  | |  | |  | |  | |
| Sesquiterpene #1 | 7.2 | | 0.15 | | | 0.70 | |  | |  | |  | |  | |  | |
| Sesquiterpene #2 | 10.5 | | 0.55 | | | 1.97 | |  | |  | |  | | 5.882 | |  | |
| Sesquiterpene #3 | 17.2 | | 3.47 | | | 8.46 | |  | | 21.368 | |  | |  | |  | |
| γ-Muurolene | 18.8 | | 2.43 | | | 5.59 | |  | |  | |  | |  | |  | |
| δ-Cadenine | 1.0 | | -0.01 | | | 0.51 | |  | |  | |  | |  | |  | |
| Germacrene B | 24.4 | | 4.87 | | | 9.84 | | 20.356 | |  | | 19.930 | |  | |  | |
| Isolongifolene | 12.3 | | -4.23 | | | 13.76 | |  | |  | |  | |  | |  | |
| Cedrene-13-ol, 8 | 0.8 | | 1.13 | | | 16.67 | |  | |  | |  | |  | |  | |
| α-Cadinol | 6.7 | | 4.37 | | | 19.03 | |  | |  | |  | |  | |  | |
| allo-Aromadendrene | 2.2 | | 2.11 | | | 17.59 | |  | |  | |  | |  | |  | |
| **BIC** |  | |  | | |  | | -723.8 | | -723.1 | | -722.9 | | -722.8 | | -722.6 | |
| **Posterior Probability** |  | |  | | |  | | 0.026 | | 0.018 | | 0.017 | | 0.015 | | 0.014 | |

Bayesian Model Averaging (BMA package in R) used to assess effect of the concentration of each individual compound

**Table S4d. Effect of concentration of stored compounds on the presence of *Myrmica rubra* ants before aphid colonisation**

| **Response:** | **Presence of *Myrmica rubra* ants before aphid colonisation (binomial)** | | | | | | | | | | | | | | | | |
| --- | --- | --- | --- | --- | --- | --- | --- | --- | --- | --- | --- | --- | --- | --- | --- | --- | --- |
|  |  | |  | | |  | | **Five best models** | | | | | | | | | |
| **Predictors** | **PEP** | | **EV** | | | **SD** | | **Model 1** | | **Model 2** | | **Model 3** | | **Model 4** | | **Model 5** | |
| Intercept | 100 | | -1.55 | | | 0.14 | | -1.519 | | -1.586 | | -1.655 | | -1.796 | | -1.539 | |
| ***Covariates*** | |  | |  |  | |  | |  | |  | |  | |  | |  |
| Plant size | 10.5 | | 0.04 | | | 0.12 | |  | |  | | 0.336 | |  | |  | |
| Accessibility | 13.2 | | 0.00 | | | 0.00 | |  | |  | |  | | 0.002 | |  | |
| ***Monoterpenes*** | |  | |  |  | |  | |  | |  | |  | |  | |  |
| β-Pinene | 0.7 | | -0.01 | | | 1.29 | |  | |  | |  | |  | |  | |
| ***Monoterpene Acetates*** | | | | |  | |  | |  | |  | |  | |  | |  |
| Verbenyl Acetate | 6.7 | | -0.96 | | | 4.33 | |  | |  | |  | |  | | -15.760 | |
| Bornyl Acetate | 53.3 | | 8.60 | | | 9.30 | |  | | 14.960 | |  | | 15.850 | | 19.240 | |
| Dihydrocarvyl Acetate | 0.7 | | 0.00 | | | 0.01 | |  | |  | |  | |  | |  | |
| Isopulegol Acetate | 3.5 | | 0.01 | | | 0.04 | |  | |  | |  | |  | |  | |
| Myrtenyl Acetate | 1.9 | | 0.03 | | | 0.28 | |  | |  | |  | |  | |  | |
| α-Terpinyl Acetate | 2.5 | | 0.27 | | | 2.24 | |  | |  | |  | |  | |  | |
| ***Oxygenated Monoterpenes*** | | | | |  | |  | |  | |  | |  | |  | |  |
| Sabinol | 1.7 | | 0.62 | | | 8.84 | |  | |  | |  | |  | |  | |
| Berbenol | 3 | | 0.39 | | | 2.74 | |  | |  | |  | |  | |  | |
| Camphenol-6 | 1.6 | | -0.09 | | | 1.66 | |  | |  | |  | |  | |  | |
| Borneol | 1.1 | | 0.04 | | | 0.62 | |  | |  | |  | |  | |  | |
| Myrtenol | 1.3 | | 0.00 | | | 0.41 | |  | |  | |  | |  | |  | |
| (*Z*)-Carveol | 0.7 | | 0.00 | | | 0.09 | |  | |  | |  | |  | |  | |
| ***Sesquiterpenes*** | |  | |  |  | |  | |  | |  | |  | |  | |  |
| γ-Elemene | 11.5 | | -12.53 | | | 40.49 | |  | |  | |  | |  | |  | |
| Sesquiterpene #1 | 2.5 | | -0.03 | | | 0.26 | |  | |  | |  | |  | |  | |
| Sesquiterpene #2 | 2.6 | | -0.07 | | | 0.63 | |  | |  | |  | |  | |  | |
| Sesquiterpene #3 | 2.7 | | -0.29 | | | 2.28 | |  | |  | |  | |  | |  | |
| γ-Muurolene | 3.3 | | -0.22 | | | 1.58 | |  | |  | |  | |  | |  | |
| δ-Cadenine | 3.2 | | -0.11 | | | 0.80 | |  | |  | |  | |  | |  | |
| Germacrene B | 2.9 | | -0.29 | | | 2.19 | |  | |  | |  | |  | |  | |
| Isolongifolene | 1.4 | | 0.03 | | | 1.13 | |  | |  | |  | |  | |  | |
| Cedrene-13-ol, 8 | 1.5 | | 0.38 | | | 7.45 | |  | |  | |  | |  | |  | |
| α-Cadinol | 95.3 | | -898.40 | | | 318.80 | | -933.0 | | -926.1 | | -974.4 | | -999.4 | | -999.1 | |
| allo-Aromadendrene | 95.3 | | 1553.00 | | | 571.30 | | 1616.0 | | 1601.0 | | 1701.0 | | 1720.0 | | 1747.0 | |
| **BIC** |  | |  | | |  | | -678.2 | | -678.0 | | -675.9 | | -675.8 | | -675.6 | |
| **Posterior Probability** |  | |  | | |  | | 0.127 | | 0.116 | | 0.042 | | 0.04 | | 0.035 | |

Bayesian Model Averaging (BMA package in R) used to assess effect of the concentration of each individual compound

**Figure S1:** Full correlation matrix of all putatively emitted compounds

**
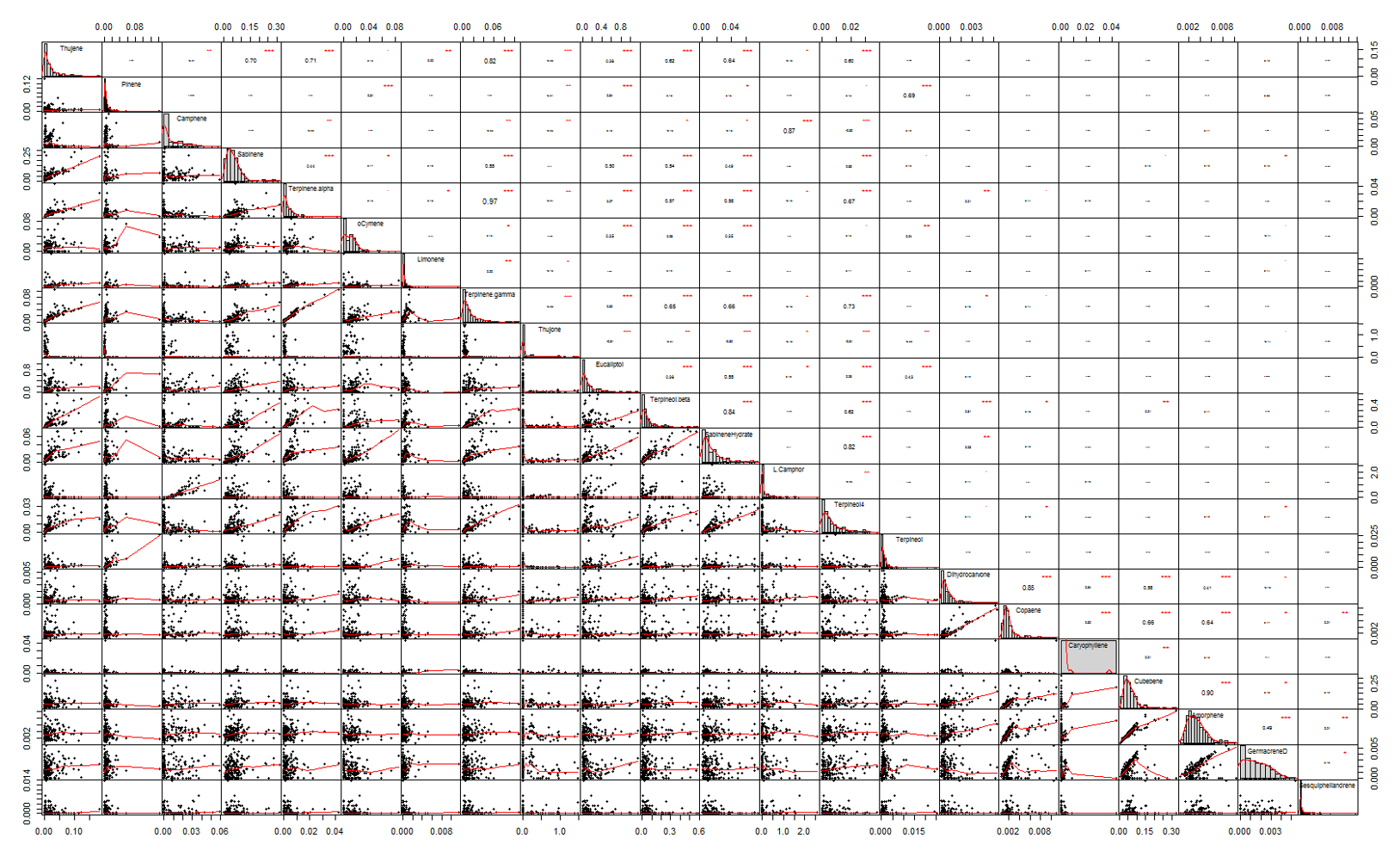
**

**Figure S2:** Map of the field site showing chemotypes. GPS coordinates were plotted using SigmaPlot version 11, from Systat Software, Inc., San Jose California USA, www.systatsoftware.com


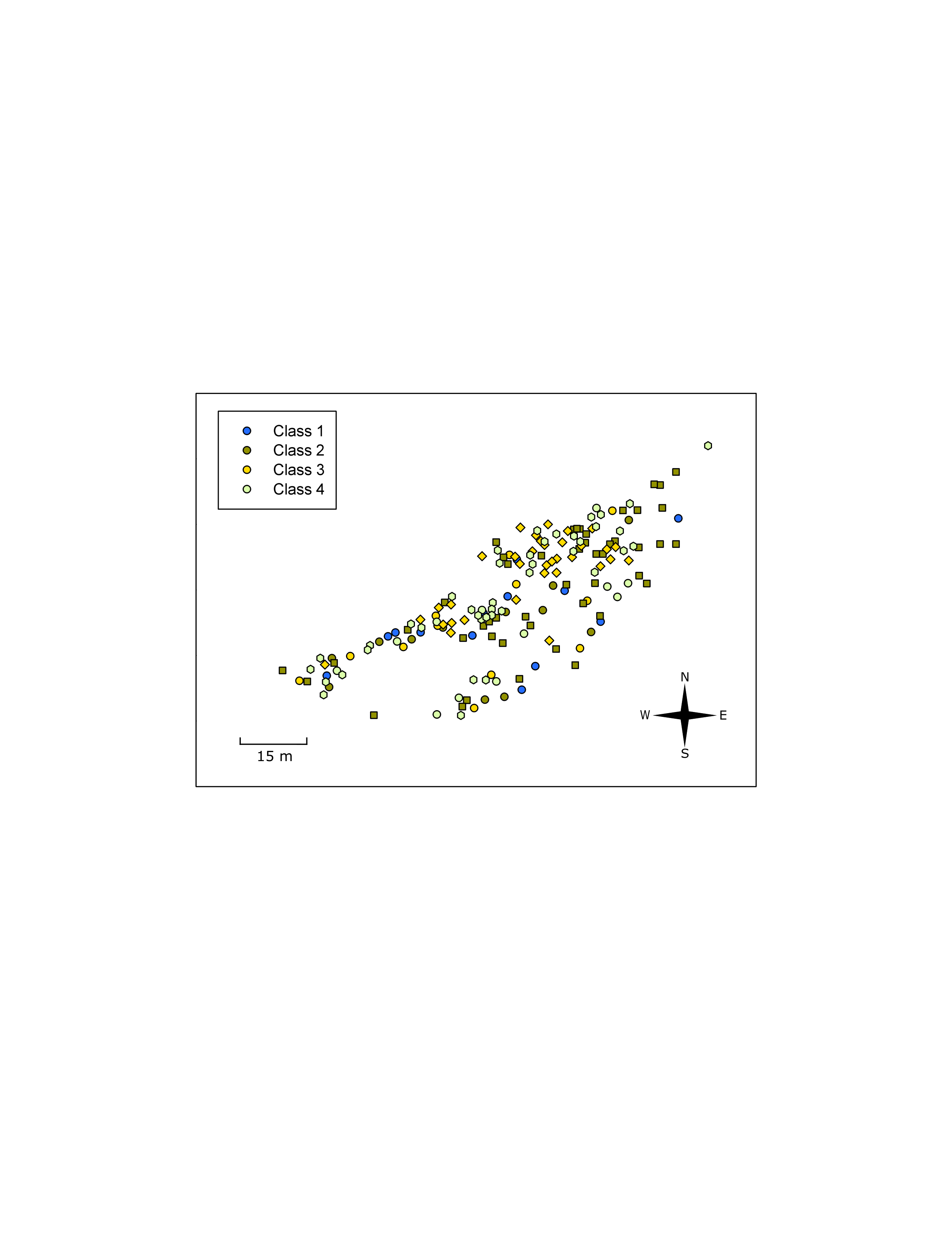

Supplement: Supplementary Data [file srep38087-s1.doc]
